# Sudden-Onset Acute Obsessive-Compulsive Disorder Associated with Streptococcus and Brain MRI Hyperintensity in a Young Adult

**DOI:** 10.3390/healthcare12020226

**Published:** 2024-01-16

**Authors:** Joan Jory, Kenneth Handelman

**Affiliations:** 1 Department of Family Relations and Applied Nutrition, University of Guelph, Guelph, ON L8N 3K7, Canada; 2 Department of Psychiatry and Behavioural Neuroscience, McMaster University, Hamilton, ON L8N 3K7, Canada; kh@cfimh.com

**Keywords:** adult, antistreptolysin, MRI, OCD, streptococcus, strep

## Abstract

Background: Pediatric autoimmune neuropsychiatric disorders associated with streptococcal (strep) infections (PANDAS) are a recognized medical entity among children. But evidence for strep-mediated sudden-onset obsessive–compulsive disorder (OCD) in young adults is very limited. Delayed strep assessment and treatment may negatively impact clinical outcomes. Methods: We describe a young adult with acute sudden-onset OCD (age 24), treated unsuccessfully with medication and therapy for 3 years. At age 27, antistreptolysin-O (ASO) was tested, based on extensive pediatric history of strep infections. Antibiotic treatment was initiated. Results: Magnetic resonance imaging (MRI) identified a new temporal lobe hyperintensity at OCD onset (age 24), which persisted at ages 25 and 30. ASO titers were elevated from age 27 through 29. Following Amoxicillin treatment, ASO initially increased. Subsequent Amoxicillin + Clavulin treatment produced improved OCD symptoms and treatment response, with no adverse effects. Conclusion: These results strongly suggest an association among strep infection, neuro-inflammation and sudden-onset OCD in this young adult whose response to medication and therapy was successful only after high-dose antibiotic intervention. Greater OCD remission potential may be possible with earlier identification and antibiotic treatment than 3 years post OCD onset. These findings add to the limited literature on strep as an etiology of the sudden-onset of OCD in young adults. They also lend urgency to increased frontline awareness for early strep and ASO assessment in sudden-onset acute OCD among young adults.

## 1. Introduction

Obsessive–compulsive disorder (OCD) can be a disabling mental illness that is estimated to affect approximately 0.93% of the Canadian population [1]. The American Psychiatric Association’s fifth edition of the Diagnostic and Statistical Manual of Mental Disorders [2] characterizes OCD as the presence of obsessions and/or compulsions, the management of which causes significant negative impacts on function, and which are not better explained by substance use, other medical conditions or other mental disorders. Tics may or may not also be present [2].

Canadians with OCD are more likely to have experienced adverse events during childhood, to have comorbid mood disorders, and to present with symptoms earlier in life [1]. Unusually, sudden-onset acute OCD has also been documented among children in relation to recent streptococcal (strep) infection [3]. This form of OCD is often associated with sudden motor abnormalities, and often involves autoimmune antibodies that target the brain [3]. The term pediatric autoimmune neuropsychiatric disorders associated with strep (PANDAS) has been coined to describe this unique infection-mediated form of acute sudden-onset OCD, and it is largely believed to be limited to the pediatric and adolescent population [3].

It is unclear, however, whether strep-mediated OCD among the young adult population is less robustly documented because it is genuinely rarer among young adults than among the pediatric population. Alternatively, a lack of awareness among frontline medical professionals and/or psychiatrists about the possibility of a strep-mediated form of acute sudden-onset OCD in young adults may exclude early testing for strep in this population, and thereby limit early strep recognition and treatment that could confer greater opportunities for OCD remission. It becomes important, therefore, to add to the evidence base for a strep-mediated form of OCD among young adults, and to raise frontline awareness of the need for strep assessment in cases of acute sudden-onset OCD symptoms in this population.

## 2. Case Report

**Figure 1:**
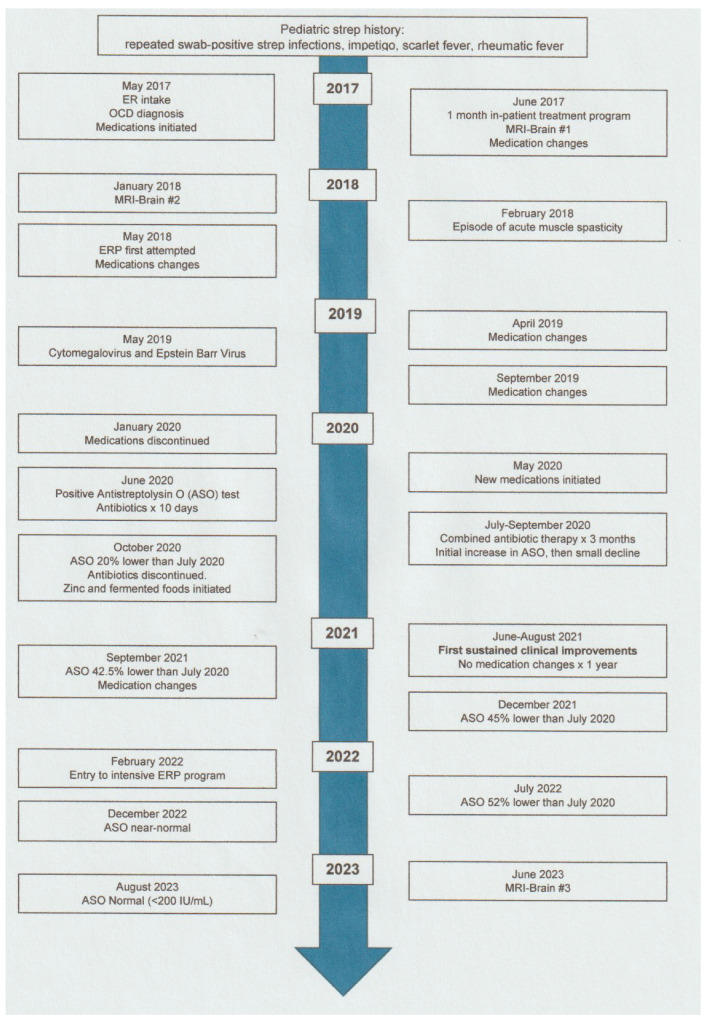
Case report timeline.

We present the case of a 24-year-old male with sudden-onset acute OCD, recurrent intrusive thoughts, anxiety, low mood and disturbed sleep following an upper respiratory infection with sore throat—a case timeline is documented in Figure 1. There was no prior personal or familial history of OCD, tics or epilepsy. Pediatric history was notable for repeated strep-positive infections, impetigo, scarlet fever, and rheumatic fever. Recent young adult history also included low immunoglobulin A (IgA: 0.7 g/mL), vitamin D deficiency (53 nmol/mL), and multiple Crohn’s-like aphthous ulcers in the distal ileum on Capsule Endoscopy.

The young man initially presented at an Emergency Department with intensely distressing thoughts, and intrusive and disturbing images, which were persistent all hours of the day and night. He identified that he wanted to die because that was the only way to escape the intensity and relentlessness of the intrusive thoughts. The patient was admitted to a secure ward for psychiatric evaluation. Severe OCD (using DSM-V criteria) was diagnosed by a psychiatrist. No tics or motor abnormalities were identified. Presentation was notable for a predominance of incapacitating obsessions; compulsions were limited, and constituted mental acts such counting and repetition; compulsions were limited, and constituted mental acts such counting and repetition. No contamination, washing, or hoarding themes were present. The patient expressed awareness that their intrusive thoughts were irrational/not true.

Drug screening was negative for all common drugs of abuse. A sore throat was noted upon hospitalization, but no strep or antistreptolysin O (ASO) testing was undertaken; ASO is an antibody against streptolysin, associated with group-A streptococcus (GAS) infections [4,5]. He was started on Quetiapine and Escitalopram, with minimal symptom response.

The patient was subsequently transferred, with consent, to a psychiatric hospital for a one-month in-patient treatment program. His Yale–Brown Obsessive-Compulsive Scale (Y-BOCS) score was 23/40 upon admission to the treatment program. For reasons that are unclear, the Y-BOCS was not re-administered during the 1-month program. Quetiapine was discontinued in favour of Escitalopram monotherapy, with the addition of benzodiazepines. The patient remained minimally treatment responsive over the 1-month period and reported no relief from the intrusive thoughts. He was discharged back into the community under general practitioner care, with support from psychiatrist e-consultations.

**Figure 2:**
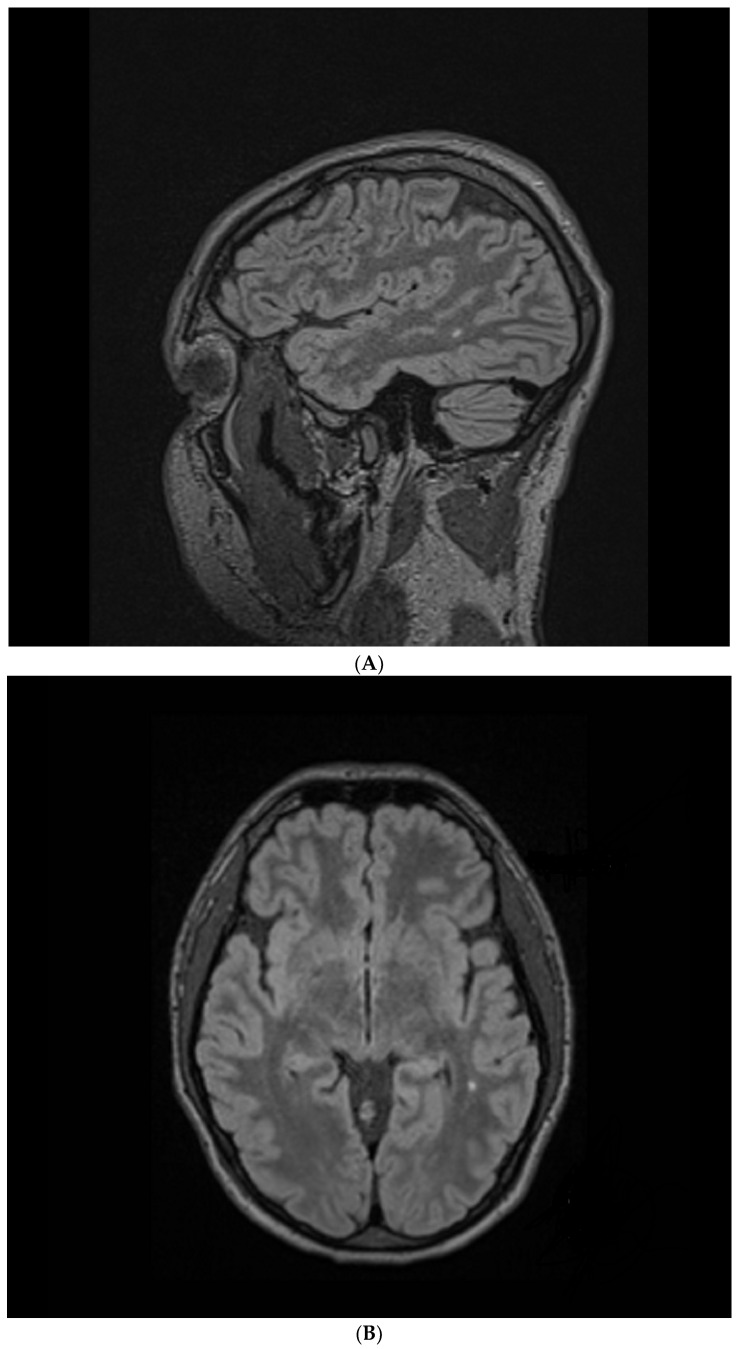
Anatomic magnetic resonance imaging (MRI) scans, using a 1.5 Tesla Siemens Avanto, without contrast. Repetition time (TR) = 6000 ms, echo time (TE) = 35.8 ms, matrix = 256 × 218 mm, number of excitations (NEX) = 186. (**A**) Sagittal Flair ISO, field of view (FOV) = 227 × 260 mm, slice thickness (SL) = 1.00 mm. (**B**) Axial Flair MPR, FOV = 260 × 260 mm, SL = 1.10 mm.

During the psychiatric treatment program, the patient was referred for a brain MRI which identified a new T2/FLAIR hyperintensity in the deep white matter of the left temporal lobe, measuring 3.3 × 2.0 mm (axial plane), of questionable clinical significance. One year later (age 25), the hyperintensity measured 3.6 × 2.3 mm (axial plane). A neurology consultant identified no neurological focal impairments, and excluded any intra-axial or extra-axial tumors. Five years later (age 30; Figure 2), the hyperintensity measured 4.0 × 3.0 mm (axial plane) and 5.4 × 3.3 mm (sagittal plane); 3-dimensional imaging was available only for the latter MRI.

The patient exhibited minimal response to multiple psychiatric medications (Table 1) and therapy modalities, both in hospital and post discharge. Adjunctive medications included: clonazepam, diazepam, lorazepam, and zopiclone. Initial medication choices were guided by pharmacogenetics; the patient is a CYP2B6 intermediate metabolizer and a CYP2D6 poor metabolizer.

One year post OCD onset (early 2018; age 25), the patient experienced several hours of involuntary muscle spasticity in his limbs, torso, and face with impaired ability to form words. He was taken to an Emergency Department (ED). The patient was held for assessment which identified no causative etiology, but strep and ASO titers were not tested.

Also in 2018 (age 25), the patient first initiated Exposure and Response Prevention (ERP) therapy. He completed 15 sessions over 6 months in 2018 but tolerated the ERP poorly due to the distress the treatment evoked and he remained treatment unresponsive.

Two years post OCD onset (spring 2019; age 26), the patient was again ill with throat symptoms. Serological testing identified co-existing Epstein Barr Virus (EBV) and Cytomegalovirus (CMV) infections but failed to include testing of strep or ASO.

Three years post OCD diagnosis (early 2020; age 27), the patient remained minimally responsive to both psychiatric medications and therapy. Frustrated with his continued lack of clinical improvement, the patient temporarily discontinued treatment.

In May 2020 (age 27), the patient again sought psychiatric assistance. A different OCD rating scale was used; his Florida Obsessive-Compulsive Inventory (FOCI) scored 12/20 on the symptom checklist and 20/20 on the severity test. He was restarted on medication, with a combination of Sertraline and Mirtazapine. ASO was tested at this time and was positive (Table 2).

Given the patient’s complex strep history, a 10-day amoxicillin course was prescribed [6,7,8]. ASO retesting (July 2020) demonstrated an unexpected increase. Anti-deoxyribonuclease-B (ADNB) antibody was negative; ADNB is also an antibody associated with GAS infections [5].

Consensus on longer term antibiotic treatment in strep-related neurological illness is limited; however, ASO titers that are initially high, then increase, suggest very recent infection with an increased risk of post-streptococcal complications including endocarditis, glomerulonephritis, and rheumatic fever [9,10,11,12]. Amoxicillin-clavulanate was therefore prescribed for 2 months; repeat testing (September 2020) demonstrated a modest ASO decrease. Amoxicillin-clavulanate treatment was continued for a third month. Repeat testing indicated a further modest ASO decline (October 2020).

Guidelines for longer antibiotic treatment for a young adult with a prior pediatric history of strep complications, including rheumatic fever and sustained young adult ASO elevations during OCD, do not exist. With antibiotic treatment, ASO levels do tend to peak approximately 3 weeks post-infection, then plateau somewhat for 6 months and slowly decline [13]. Therefore, no further antibiotic treatment was undertaken. Instead, the patient initiated supplementation with 30 mg/day zinc bisglycinate and increased consumption of fermented foods to support post-antibiotic gastrointestinal health.

Notable improvements in OCD symptomology were identified in summer 2021 (age 28; 4 years post OCD onset), when ASO titers had declined by approximately 50%. By contrast, medications and treatment had remained unchanged for more than 1 year (May 2020).

By early 2022 (age 29; 5 years post OCD onset), the patient’s ASO titers were dropping towards 50% of their highest level (440 IU/mL in July 2020), and were approaching normal (<200 IU/mL). For the first time since OCD onset, he felt able to enter an intensive ERP treatment program (full days, 5 days/week × 6 weeks); the patient had previously been unable to successfully complete ERP due to the psychological distress the treatment evoked while his ASO titers were elevated. Again, a different OCD rating scale was employed. His Obsessive-Compulsive Inventory—Revised (OCI-R), which correlates well with Y-BOCS and FOCI scales [14,15], was 22/72 in March 2022; levels > 21 are considered clinically positive for OCD.

Six years post OCD onset (August 2023; age 30), the patient’s ASO titer was normal for the first time in 3 years. Though not in clinical remission [16], the patient subjectively identifies as having good functionality and is in the final stages of an advanced graduate degree. There have been no symptom regressions or exacerbations since summer 2021; although, the patient is experiencing hypertriglyceridemia and hyperinsulinemia effects attributed to the use of sertraline [17,18] and clomipramine [19].

## 3. Discussion

### 3.1. Strep and OCD in Young Adults

**Figure 3:**
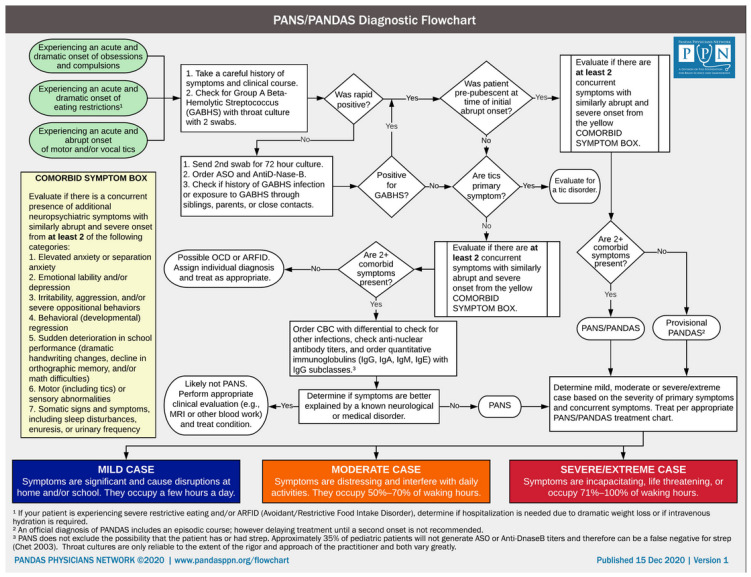
PANS/PANDAS diagnostic flowchart. Reprinted with permission from the PANDAS Physicians Network [25]. Copyright © 2024, PANDAS Physicians Network (PPN).

The association between GAS infections and OCD in the literature has largely been focused on the childhood population, and PANDAS (pediatric autoimmune neuropsychiatric disorders associated with strep) and PANS (pediatric acute-onset neuropsychiatric syndrome) specifically [20,21,22,23]. The diagnostic criteria for these infection-mediated neuropsychiatric disorders have been well codified by Swedo et al. [24] (Table 3) and the PANDAS Physicians Network [25] (Figure 3). Based on these codifications, the young adult patient in our report appears to fulfill all the criteria for PANDAS neuropsychiatric illness presenting as OCD, except prepubertal onset. The temporal association between GAS and symptom onset for this patient is inferred based on the presence of a sore throat upon admission; strep and/or ASO were not tested at symptom onset. The subsequent symptom improvement after post-antibiotic ASO decline, with no change in medication or therapy for nearly a year, lends further support to a temporal association between GAS and symptoms. Swedo et al. [24] note that it can often be difficult to definitively establish a preceding GAS infection [25].

In contrast to the substantial evidence base for strep-mediated OCD in children, there are very few published reports of young adults with strep-mediated OCD [26,27,28]; more specifically, there are no case reports documenting confirmed strep-positive history in childhood, sustained elevations of ASO titers in young adult OCD patients, and temporally related MRI abnormalities.

One report, by Deshmukh et al. [27], reported sudden-onset OCD with recurrent thoughts in a 22-year-old male 4 days after a high fever and sore throat. His history was only suggestive of prior strep infections, but his ASO titers tested positive at >200 IU/mL. No brain lesion was identified. Notably, the patient experienced complete OCD remission following a 7-day Azithromycin treatment initiated very shortly after OCD onset. This raises the possibility that early identification of elevated ASO titers and/or active strep infection may have important implications for successful clinical outcomes.

A second report, by Endres et al. [29], identified severe sudden-onset OCD in a female young adult with a left periventricular MRI abnormality of presumed autoimmune inflammatory origin. Antineuronal antibodies were negative but cerebrospinal fluid (CSF) analysis was consistent with an autoimmune inflammatory process. Treatment with 7 weeks of immunotherapy, pulse steroids, and ERP therapy was rapidly initiated. Following treatment, the patient’s Y-BOCS had completely normalized (from 29 to 0) and their MRI lesion had regressed. In this case, the early detection and intensive multi-modal treatment clearly made an important contribution to the full clinical remission experienced by this young adult.

By contrast, Bodner et al. [30] documented sudden-onset OCD in a 25-year-old male following a severe sore throat whose positive ASO titers were only identified 3 years post OCD onset. The patient presented with a right globus pallidus MRI hyperintensity and positive anti-neuronal antibodies. This is one of the earliest cases to document a PANDAS-like relationship between strep and young adult sudden-onset OCD, but no information was provided on the patient’s subsequent treatment or their treatment response.

Like the Bodner case, the patient in this report also experienced a 3-year delay in the detection of elevated ASO despite presenting with throat symptoms at initial OCD onset and hospitalization, presenting with throat symptoms during a subsequent EBV and CMV illness, and presenting in ER with an episode of pronounced motor spasticity.

Like the Bodner case, the current patient also presented with an MRI abnormality. Berthier et al. [31] employ the term ‘acquired OCD’ for OCD patients with confirmed focal brain lesions, including those in the temporal lobe. Patients with ‘acquired’ OCD are more likely to have a negative family history and to have a later age of OCD onset [31], consistent with the current patient’s presentation.

The current patient’s temporal lobe hyperintensity, though unilateral, resembles MR imaging of limbic encephalitis (LE) [32], an antibody-mediated neuro-inflammatory process with a range of psychiatric symptoms [33]. Strep has been identified as an etiological vector of neuro-inflammatory LE [34,35]. Testing anti-neuronal antibodies can identify neuro-inflammatory processes in both strep-positive and ASO-positive young adult OCD [36]. The ‘Cunningham Panel’ of anti-neuronal antibodies has demonstrated good correlation with changes in neuropsychiatric symptoms in PANDAS [26,37]. However, anti-neuronal antibody testing is minimally available in Canada [38], despite an emergent awareness of strep–OCD relationships among Canadian practitioners [39]. Limitations on anti-neuronal antibody testing in Canada also limits the evidence base for initiating potentially therapeutic pulse steroids, immunotherapy, and/or plasmapheresis treatment in our young adults presenting with sudden-onset debilitating OCD, ASO-positivity, and MRI hyperintensities.

### 3.2. Gut Health and Strep Susceptibility

It is of note that this patient was also diagnosed with Crohn’s-like aphthous ulcers, 5 years preceding OCD onset. Up to 50% of Crohn’s Disease (CD) patients have elevated ASO, and ASO positivity has been associated with new-onset CD [4]. Both PANDAS and CD are also associated with altered gut microbiota [40]. Common etiological factors, including infection and altered microbiota, may impact both the risk of new onset OCD and of Crohn’s. This is supported by the emerging relationship between COVID-19 and new-onset inflammatory bowel disease [41,42,43,44].

Following completion of their antibiotic treatment, this patient introduced zinc supplementation to support their gastrointestinal health. Zinc has antiviral properties and a symbiotic relationship with gut microbiota [45]. Zinc deficiency can occur in 21–50% of CD patients [46,47,48], and zinc status can be inversely associated with CD [47]. Zinc supplementation improves therapeutic outcomes among CD adults [49].

Zinc also has antimicrobial effects on both Group B [50] and A [51,52] streptococcus. GAS competes for zinc with the host defense systems and can contribute to post-infection zinc deficiency [53]. It is possible, therefore, that this patient’s zinc supplementation may have contributed to their post-antibiotic ASO improvements, while perhaps also indicating a post-infection decline in zinc status that might otherwise have rendered them more susceptible to future strep infections.

In addition to zinc, this patient increased their intake of fermented foods after antibiotic cessation. Emerging evidence suggests fermented foods may have important antimicrobial and immune-modulating benefits [54,55,56,57]. Increased fermented food consumption in this case could potentially have conferred additional antimicrobial benefits, possibly through supporting a microbiota environment less hospitable to future GAS involvement.

Whether or not the zinc and fermented foods, in addition to the antibiotic treatment, contributed directly to the reduction in antistreptolysin titers would be difficult to establish. However, it is plausible that continued supplementation with zinc and fermented foods may have conferred protection against future strep infections in this patient with a very strong history of strep-related illness, given that there have been no further documented strep infections or OCD exacerbations since the zinc and fermented foods were initiated.

### 3.3. MRI Interpretation

The sequential MRIs have documented a small incremental increase in the temporal lobe intensity from 2017 to 2018, and again between 2018 and 2023. Since antibiotic treatment had not yet been initiated in 2018, the first incremental increase may potentially be explained by an ongoing strep-mediated inflammatory process.

Antibiotic treatment was initiated in summer 2020. Since this was the first year of the COVID-19 pandemic, access to medical services including MRI was severely restricted globally. A follow-up MRI was not obtained until 3 years after initiation of antibiotic treatment, and was completed only after the patient’s antistreptolysin levels were near normal in summer 2023.

It is possible that the second interval increase in the temporal lobe hyperintensity, between January 2018 and June 2023, reflects 2 years (2018–2020) of persistent inflammation prior to antibiotic treatment, followed by a slow but incomplete decrease in inflammatory effects as antistreptolysin levels slowly declined. In other words, it is possible that there was a much greater interval increase in temporal lobe hyperintensity in 2020, which cannot be confirmed because no MR imaging was possible at that time, followed by a gradual decrease in temporal lobe inflammation as the ASO titers normalized.

Follow-up MRIs will help to elucidate whether a subsequent decline in temporal lobe hyperintensity is possible, and whether any potential decline in hyperintensity overlaps with further clinical improvements for this patient whose OCD is stable and well managed but not in remission. In other patients with sudden-onset strep-related OCD, immediate treatment has led to complete remission including of MRI abnormalities [21,23]. However, it is also possible that the 3 year delay in ASO testing and antibiotic treatment experienced by this patient may have conferred more permanent limitations on their potential to resolve their temporal lobe hyperintensity, despite having now normal ASO levels, and on their potential to achieve long-term disease remission.

### 3.4. Psychiatric Medication

Use of the tricyclic antidepressant (TCA) clomipramine is not recommended for CYP2D6 poor metabolizers [58]. The selective serotonin reuptake inhibitor (SSRI) sertraline is partially metabolized via CYP2B6, so adverse effects may be increased with CYP2B6 intermediate metabolizers [59]. Additionally, SSRIs can increase serum clomipramine levels, and clomipramine can increase SSRI absorption and binding [60]. Thus combined sertraline and clomipramine treatment was initiated only after other medications less impacted by altered CYP2B6 and CYP2D6 pathways had been trialed.

The patient has experienced hypertriglyceridemia and hyperinsulinemia, which emerged only after the initiation of combined high-dose sertraline and clomipramine, consistent with documented medication effects. Sertraline is associated with increased insulin and triglyceride levels [17] and altered mitochondrial glucose handling [18]. Clomipramine is associated with hyperglycemia [19]. Clomipramine is also associated with potentially fatal QT prolongation and other cardiac abnormalities [61]. The patient remains under physician care and is being monitored.

Notably, both clomipramine and sertraline are also associated with significant alterations in gut microbiota [62], and sertraline may significantly increase the risk of antibiotic resistance [63]. Given the emerging relationships of zinc and fermented foods with gut microbiota and microbial resistance discussed earlier, the concurrent use of zinc and fermented foods during combined clompramine and sertraline treatment may confer an important protective advantage against these additional adverse medication effects.

### 3.5. Limitations

The absence of strep or ASO testing upon sudden OCD onset is a limitation to establishing a conclusive link between strep and OCD in this case report. However, the numerous missed testing opportunities for this patient despite several early symptomatic encounters with the medical system also serve to underscore the urgent need for change.

The use of different OCD symptom scales is a potential limitation. However, it also appropriately reflects the reality of a patient navigating the mental health care system which often entails encounters with a variety of hospital, clinical, and practitioner settings, each of whom may use a different scale.

Uniquely, the early medication choices in this case report were initially guided by pharmacogenetics; the use of non-traditional OCD medications could otherwise be considered a limitation. However, it is notable that even sustained high doses of traditional OCD medications in the later years of treatment failed to have a clinically therapeutic effect until after the antibiotic-mediated decline in the patient’s ASO titers. Only when the ASO titers were near normal was the patient sufficiently stable to tolerate ERP therapy, and the combination of lower ASO, high-dose OCD medication, and effective ERP psychotherapy led to significant clinical improvement.

## 4. Conclusions

We present the first report of sudden-onset acute OCD in a young adult Canadian with recurrent strep-positive infections in childhood, sustained young adult ASO elevations, and temporal lobe MRI hyperintensity. Although strep causality cannot be definitively proven, the strong pediatric history of strep-positive infections, the sustained ASO elevations through acute phases of young adult OCD presentation, and the improvements in both pharmaceutical and psychological treatment response as ASO levels declined are highly suggestive of an association.

Overall, our findings, which merit further investigation, contribute new insights to a slowly emerging evidence base for an infective and/or neuro-inflammatory etiology in the pathogenesis of sudden-onset acute OCD in young adults. Notably, the few published case reports to date highlight considerable heterogeneity in timely access to etiological testing and treatment, with variable impacts on OCD recovery.

Increased frontline physician and hospital awareness of the need for early testing of strep and ASO, as well as neuro-inflammation biomarkers where available, should be considered a priority, as delayed diagnosis may have important negative implications for treatment options and remission potential among young adults with sudden-onset acute OCD unaccompanied by prior OCD history. Improved clinical outcomes and OCD remission potential associated with early infection diagnosis among even a small group of young adult OCD patients could improve illness and quality of life and reduce costs for both the patient and publicly funded health care systems.

Further investigation into the possible roles of zinc and fermented foods in mediating strep susceptibility and/or modifying post-antibiotic relapse potential, particularly among patients with co-existing gastrointestinal disorders, should be considered. Their capacity to offset potentially serious medication-induced alterations to microbiota and antibiotic resistance also merits exploration.

## Figures and Tables

**Table 1:** Chronology of OCD medications.

Dates	Age (Years)	Medications	Maximum Dose
June 2017	24	Escitalopram + Quetiapine	10 mg 50 mg
July 2017–May 2018	24–25	Escitalopram monotherapy	40 mg
May 2018–April 2019	25–26	Venlafaxine XR monotherapy	262.5 mg
April–September 2019	26	Desvenlafaxine + Lurasidone	100 mg 40 mg
September–December 2019	26	Lurasidone monotherapy	40 mg
January–April 2020	26–27	Patient discontinued OCD medications	0
May 2020–September 2021	27–28	Sertraline + Mirtazapine	400 mg 45 mg
September 2021–current	28–30	Sertraline + Clomipramine	400 mg 50 mg

**Table 2:** Antistreptolysin (ASO) and anti-deoxyribonuclease-B (ADNB) results.

Collection Date	Age (Years)	ASO Titer Normal: <200 IU/mL	ADNB Titer Normal: <301 U/mL
June 2020	27	416	-
July 2020	27	440	<95
September 2020	27	396	-
October 2020	27	360	-
September 2021	28	253	-
December 2021	28	241	-
July 2022	29	211	-
December 2022	29	202	-
August 2023	30	124	-

**Table 3:** Diagnostic criteria for PANDAS and PANS.

	PANDAS Diagnostic Criteria
Criteria	Description
1.	Presence of obsessive–compulsive disorder (OCD) or tic disorder
2.	Prepubertal symptom onset
3.	Acute symptom onset and episodic course
4.	Temporal association between Group A streptococcal infection and symptom onset/exacerbations
5.	Associated with neurological abnormalities (particularly motoric hyperactivity and choreiform movements)
	**PANS Diagnostic Criteria**
Criteria	Description
I.	Abrupt, dramatic onset of obsessive–compulsive disorder or severely restricted food intake
II.	Concurrent presence of additional neuropsychiatric symptoms, with similarly severe and acute onset, from at least two of the following seven categories:
	1. Anxiety
	2. Emotional lability and/or depression
	3. Irritability, aggression an/or severely oppositional behaviours
	4. Behavioural (developmental) regression
	5. Deterioration in school performance
	6. Sensory or motor abnormalities
	7. Somatic signs and symptoms, including sleep disturbances, enuresis or urinary frequency
III.	Symptoms are not better explained by a known neurologic or medical disorder, such as Sydenham chorea, systemic lupus erythematosus, Tourette disorder or others.

Adapted with permission from Swedo SE et al. [25] under the terms of the Creative Commons Attribution License. Copyright © 2024, [25].

## Data Availability

Data are contained within the manuscript.
